# The prenatal diagnosis and genetic counseling of chromosomal micro-duplication on 10q24.3 in a fetus: A case report and a brief review of the literature

**DOI:** 10.1097/MD.0000000000022533

**Published:** 2020-10-16

**Authors:** Shaoyang Lai, Xueqin Zhang, Ling Feng, Mengzhou He, Shaoshuai Wang

**Affiliations:** a Department of Obstetrics, Women and Children's Hospital, School of Medicine, Xiamen University, Xiamen; b Department of Obstetrics and Gynecology, Tongji Hospital, Tongji Medical College, Huazhong University of Science and Technology, Wuhan, China.

**Keywords:** 10q24.3, chromosome micro-duplication, genetic counseling, prenatal diagnosis, Split-hand/split-foot malformation

## Abstract

**Rationale:**

Split-hand/split-foot malformation (SHFM), also known as ectrodactyly, is a congenital limb malformation affecting the central rays of the autopod extending to syndactyly, median clefts of the hands and feet, aplasia/hypoplasia of phalanges, metacarpals and metatarsals. Duplication of this 10q24 region is associated with SHFM3. While the clinical and genetic heterogeneity of SHFM makes the prenatal diagnosis and genetic counseling more challenging and difficult.

**Patient concerns:**

A physically normal pregnant woman had a systemic ultrasound at the second trimester, only identified the deformity of both hands and feet on the fetus.

**Diagnoses:**

The fetus was diagnosed as sporadic SHFM3.

**Interventions:**

After seeking advice from genetic counseling, she decided to terminate the pregnancy. The induction of infant was done after appearance of bipedal clefts, lobster-claw appearance and partial loss of phalanges and metacarpals, leaving behind 2nd finger in the left hand and the 5th in the right hand. Furthermore, collection of umbilical cord is recommended to this fetus for genome-wide detection.

**Outcomes:**

An outcome of the gene detection from abortion shows that there is variation in copy number in genome of chromosome 1 and chromosome 10.

**Lessons:**

This case study confirms an association between SHFM3 and chromosomal micro-duplication on 10q24.3, and the extension of clinical spectrum of SHFM3. It also proposes some prenatal diagnosis and genetic counseling to help in planning and management in affected pregnancy. This will reduce the congenital and development abnormalities in birth rate, as well as relive the economic, psychological, and physical burden to the affected families.

## 1 Introduction

Genomic imbalances are major causes of congenital abnormalities which are lifelong disability resulting to significant economic, psychological and physical burden to the affected families, society affected and government in general. Congenital abnormalities affect 5% of live births,^[1]^ an average of 300,000 newborns die within 4 weeks after birth annually due to congenital defects globally.^[2]^ Some of these genetic anomalies resulting from phenotypes are numerous and some of them are associated with chromosomal deletions and duplication.

Split-hand/split-foot malformation (SHFM), also known as ectrodactyly; it's one of the most complex human congenital limb malformations affecting the central rays of the autopod involving syndactyly, median clefts of the hands and feet, aplasia/hypoplasia of phalanges, metacarpals and metatarsals. This is a rare condition that occurs in 1 out of 8500 to 25,000 newborns that accounts for about 15% of all limb reduction defects.^[3]^ A great number of gene defects can cause SHFM. Currently, seven known loci for SHFM have been mapped in human genome: SHFM1 (7q21.3), SHFM2 (Xq26), SHFM3 (10q24.3), SHFM4 (3q27), SHFM5 (2q31), SHFM6 (12q13.11-q13) and SHFM/SHFLD (17p13.3).^[4]^ SHFM is heterogeneous disorders which shows variable degree of phenotypes between families, inter-individual and even amongst limbs of a single individual, ranging from syndactyly, oligodactyly to monodactyly_._^[5]^ This presents in both non-syndromic and syndromic forms: non-syndromic forms may occur as an isolated entity; and syndromic ones is always associated with other anomalies. Both these two forms can be familial or sporadic, representing all possible types of inheritance. The most common mode of inheritance is autosomal-dominant, while autosomal-recessive and X-linked forms occurs rarely.^[6–8]^

The case reports and genetic studies of familial type are most common, while that of sporadic SHFM remains limited. This case study focuses on a sporadic case which is about a chromosomal duplication on 10q24.3 in a fetus. The aim of the study is to provide clinical and molecular information about this abnormality as well as discuss the underlying pathways and mechanism that contribute to their development. Furthermore, the study will propose some prenatal diagnoses that are helpful in the planning of molecular genetic tests which is aimed at identifying disease causing mutation. The study is also going to emphasize on the significance of genetic counseling, especially in sporadic SHFM cases which will be significant to parents who decide whether to terminate the pregnancy or not, as this will minimize congenital and developmental abnormalities experienced during birth, and relive the economic, psychological and physical burden to families affected.

## 2 Case presentation

**Figure 1:**
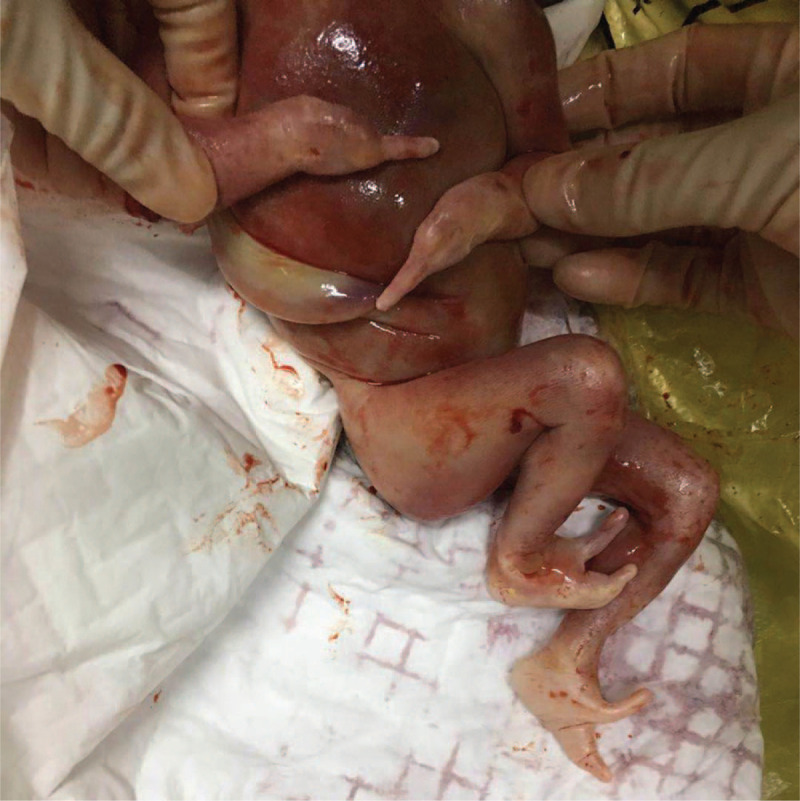
The appearance of this fetus after the induction of labor by Rivanol. The fetus presented with bipedal clefts, lobster-claw appearance, partial loss of phalanges and metacarpals, leaving only the 2nd finger in the left hand and the 5th in the right hand.

**Figure 2:**
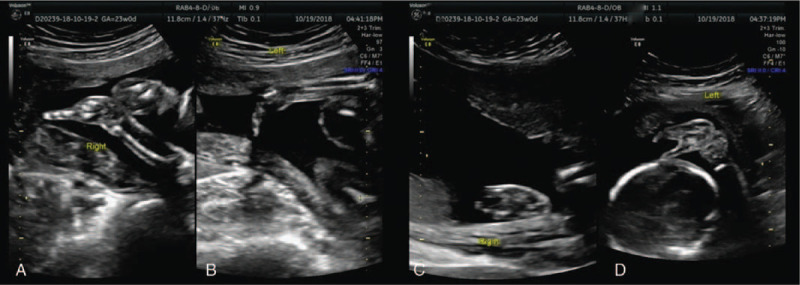
The systemic ultrasound examination of this fetus at the second trimester. It presented with deformed hands and feet. In hands, aplasia of the phalanges and metacarpals was seen, leaving the left hand only with the 2nd finger(B), the right hand only with the 5th finger(A). Both the feet had deep midline cleft, syndactyly, and aplasia of the some digits, absence of phalanges and metacarpals, giving a characteristic lobster-claw appearance (C, D).

A pregnant woman aged 29-year-old, with Gravida 1 and Para 0 was first in birth order. She had a non-consanguineous marriage. There was no history of similar malformations in these couples, as well as their sibling, or close relatives. Physical and systemic examination of the fetus in the first trimester appeared unremarkable. However, at 23rd week of gestation, systemic ultrasound showed that the fetus presented with deformed hands and feet. In hands, aplasia of the phalanges and metacarpals was seen in Figure 2, the left hand only with the 2nd finger, the right hand only with the 5th finger. Both feet had deep midline cleft, syndactyly, and aplasia of the some digits, absence of phalanges and metacarpals, giving a characteristic lobster-claw appearance (Fig. 2). Furthermore, studies on molecular genetic could not be done due to economic constraints of her family. After genetic counseling, she finally decided to terminate the pregnancy. After induction of labor by Rivanol, she labored for dead infant at 24th week of gestation period with appearance of bipedal clefts, appearance of lobster-claw and partial loss of phalanges and metacarpals, leaving only the 2^nd^ finger in the left hand and the 5th in the right hand (Fig. 1), which was consistent with the results of systemic ultrasound. The samples were collected and isolated from the umbilical cord of this fetus; the chromosome aneuploidy and the copy number variation of the genome above 100 Kilobyte were detected by high-throughput DNA sequencing.

**Figure 3:**
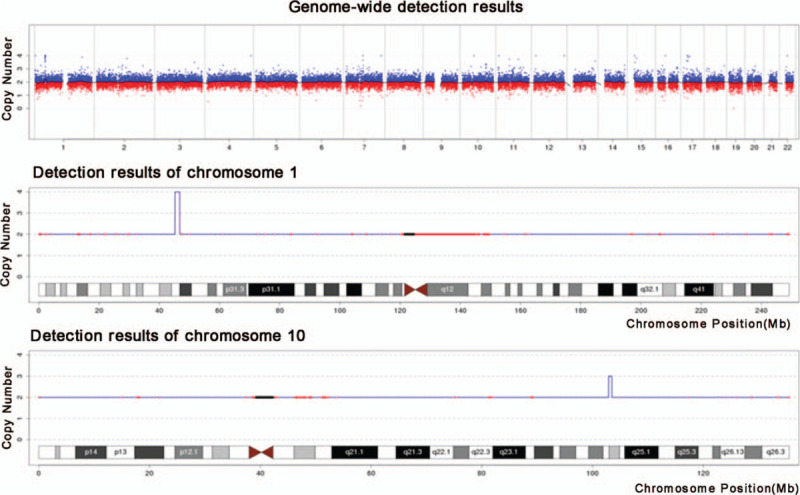
The genome-wide detection results of this fetus. It indicated that seq[hg19]dup(10)(q24.31-q24.32) (Chr10:g.102900000–103500000dup) on chromosome 10; and seq[hg19] dup(1)(q34.1p33) (Chr1:g.45220000–46820000dup) on chromosome 1.

The chromosomal findings indicated that seq [hg19] dup (10) (q24.31-q24.32) (Chr10:g.102900000–103500000dup), which means that 0.60 M chromosome region; 10q24.31-q24.32 was duplicated, covering about 7% of the regions of SHFM3; and seq [hg19] dup (1) (q34.1p33) (Chr1:g.45220000–46820000dup). There was no clear pathogenic information and reports related to the fragments (Fig. 3). Therefore, the fetus was diagnosed as sporadic SHFM3 with a distinct gene duplication syndrome.

## 3 Discussion

Several disorders have been reported to be associated with a submicroscopic duplication, deletion or inversion of genome.^[9–11]^ These disorders are categorized as being genomic disorders in contrast to classic Mendelian diseases.^[9]^ SHFM is a limb malformation characterized by median clefts, ectrodactyly, maldevelopment of the metacarpals/ metatarsals and syndactyly. SHFM is thought to originate from abnormal developmental signals during limb morphogenesis. Although, its developmental basis has not been fully elucidated, SHFM thought to be as a result of a failure to maintain the central portion of the apical ectodermal ridge (AER) within the developing autopod.^[12]^ SHFM is also heterogeneous condition that can be caused by a mutation in any point from a number of genes and loci. At least seven distinct genetic loci have been implicated from the isolated SHFM: SHFM1 at 7q21, SHFM2 at Xq26, SHFM3 at 10q24, SHFM4 at 3q27, SHFM 5 at 2q31, SHFM6 at 12q13 and SHFM/SHFLD at 17p13.^[4]^

Approximately 20% of SHFM are caused by the rearrangements at SHFM3 locus.^[5]^ In humans, duplication of this 10q24 region is associated with split hand–foot malformation (SHFM3) with a high phenotypic variability and reduced penetrance. In 1995, Nunes indicated that the locus of SHFM3 was mapped to chromosome 10q24–q25.^[13]^ In 2003, de Mollerat *et al.* showed that submicroscopic chromosomal duplication within the SHFM3 locus are associated with non-syndromic SHFM in familial and sporadic cases.^[4]^ The previous findings indicated maximum duplication to be found in at least 6 genes: LBX1 (ladybird homeobox 1), BTRC (â- transducin repeat containing), POLL (polymerase (DNA directed), lambda) and a portion of DACTYLIN (FBXW4, F-box and WD repeat domain containing 4). The minimal duplicated ones, which is approximately 325 Kilobyte in size, included only two genes, BTRC and POLL.^[14]^

However, SHFM displays highly variable phenotypes between families/individuals. Thus such a disturbed function of genes in the locus, the over-expression or a combination of one of these genes, may be due molecular defect underlying this limb anomaly. Up to date, the mechanism by which the 10q24 genomic aberration causes abnormal limb development remains elusive. Several genes within the duplication, near the duplication and insertion points are very good candidates that lead to limb malformation.

In the past decade, enormous information from clinical and bench research has led to improved understanding of molecular and biological mechanisms underlying SHFM. The best known gene is TP63, mutations that are associated with both syndromal and non-syndromal SHFM.^[15,16]^ Expectedly, the search for TP63 binding sites revealed locations within 300 Kilobyte of genes with several other SHFM loci, which suggests that these sites might consist of the regulatory elements which contributes to SHFM.^[15,16]^ FBXW4 is a member of the F-box-WD40 gene family, whose members encode subunits of ubiquitin ligases that present phosphorylated protein which targets ubiquitin-containing enzymes for degradation. The protein is thought to play a role in maintaining the AER for developing limb bud.^[17,18]^ Both LBX1 and TLX1 are HOX genes which plays the key roles in the proximal-distal axis of the limb skeleton.^[19]^ LBX1 is highly expressed in the central nervous system and skeletal muscles, this is thought to connect with migratory of muscle precursors as it maintain the potential.^[20,21]^ TLX1 also called HOX11, which encodes T-cell leukemia homeobox protein 1. TLX1 is highly expressed in zeugopod region and is used to maintain normal development of ulna and radius.^[22]^ BTRC plays a role in ubiquitination factor of proteins which is involved in various signaling transduction pathways such as Wnt/â-catenin, Sonic hedgehog and NF-êB.^[23]^ For example, the duplication of BTRC in SHFM3 may result to lower levels of â-catenin in the AER and also leads to ectrodactyly. BTRC is also involved in the NF-êB pathway; these are two other genes within the SHFM3 region: IKKa and NF-êB2. Correction of NF-êB signaling is essential for AER maintenance and limb development.^[24]^ The targeted deletion of IKKa in mice as an inhibitor of NF-êB, leads to limb and skin abnormalities.^[25,26]^ BTRC, as part of the ubiquitin protein ligase complex, it targets IKKa for destruction which eventually leads to in activation of the NF-êB pathway. Over-expression of BTRC would be predicted to reduce IKKa levels and contribute to the SHFM3 phenotype. All these pathways are involved in limb development, thus dysregulation of BTRC expression in a dosage dependent manner might be associated with SHFM3 phenotype.^[4]^ FGF8 which is also located within the SHFM3 critical region induces and regulates the limb bud patterning via AER signaling. FGF8 mouse ortholog is highly expressed throughout the AER. Its inactivation in early limb ectoderm causes hypoplasia/aplasia of specific distal skeletal elements and alters the expression of SHH and BMP2 genes.^[27]^

Gene–gene interactions might aid in the explanation of penetrance of the SHFM phenotype, as it occurs within the Dactylaplasia (Dac) mouse. A spontaneous murine model recapitulating SHFM, and studies of SHFM families also support the possibility of complex inheritance.^[28,29]^ SHFM-related genes are likely to be involved in complex networks interactions with one another. Dac is an inherited limb malformation in mice characterized by the absence of central digits, hypoplasia/aplasia of metacarpal/metatarsal bones and syndactyly.^[30]^ Both human and mouse SHFM3 regions (Dac locus maps to chromosome 19) share a high degree of homology. Thus Dac mouse is considered to be an animal model for human SHFM3. The mechanism that underlies the loss of digits in Dac mutants involves increased cell death in a specific portion of the AER. The Dac mouse model suggests that identification of the mouse and human modifier genes will shed light on the pathogenesis of SHFM related to this locus and the penetrance and variability of the phenotype.

SHFM is genetically heterogeneous with seven loci mapped to date. With consideration of reduced penetrance, variable expression or non-Mendelian inheritance as well as distortion segregation and sex bias, the over-transmission affects the genetic alteration from fathers to sons.^[5]^ It's clear that prenatal diagnosis and genetic counseling in SHFM cases is difficult and challenging, which is present not only in sporadic but also in familial cases. Furthermore, many of the SHFM cases seem to originate from complex set of mutations/chromosomal aberrations that must be viewed as two- or multigenic disorders. Consequently, identification of this genetic alteration responsible for SHFM in individual patients is of practical significance for the entire family. If the genetic counseling in SHFM is to be reliable and informative it should be based on the panel of relevant genetic testing. In this manner, one can provide an insight that is essential for developmental genes and assists in both direct mapping efforts and target genetic testing, eventually providing more accurate information for family members.

The diagnosis of patient with SHFM should be based on both careful clinical examination and relevant cytogenetic/molecular tests. Throughout the pregnancy, routine ultrasonography screening plays an important role in screening of genetic markers for example NT thickening and nasal bone loss in the first trimester, neck skin fold thickening, long bone shortening, intraventricular bright spots, and choroidal cysts in the second trimester. Continuous sequential tracking of ultrasound is required and deemed essential. In the second trimester, fetal organs should also be screened systematically by ultrasound examination. The detection rate of fetal malformation by prenatal ultrasound in the second trimester of pregnancy is about 50% to 70%.^[31]^ Then amniotic fluid, villus and fetal cell culture are used to make prenatal diagnosis of chromosomal or genetic diseases using chromosome karyotype and molecular biology methods. Subsequently, treatment and intervention to the deformed fetus will be made.

In the literature review, it is suggested that a series of diagnostic approach, such as cytogenetic/molecular tests, should be available when planning the genetic diagnostic of SHFM that is according to the relative frequencies of different causes of SHFM. For non-syndromal SHFM and SHFLD, the first diagnostic step must involve the use of a high resolution chromosome analysis and genome-wide array CGH (aCGH). Many cases are due to chromosome rearrangements or specific genomic micro-duplication. The aCGH would be the best method of choice as it allows not only for the detection of these changes, but also for the identification of other unbalanced chromosomal aberrations such as submicroscopic rearrangements affecting different loci. With the vast majority of SHFM related aberrations involve around several hundred Kilobyte of genomic DNA, the resolution of aCGH platforms does not have high. Routine diagnostic arrays with the resolution of about 180K oligonucleotide probes per haploid genome are sufficient for identifying most of the underlying copy number variations. Secondly, another important diagnostic test in SHFM patients is the TP63 gene sequencing. This is because point mutations in this gene give rise to about 10% to 16% of isolated SHFM and may occur either as de novo or have autosomal dominant inheritance with a 50% recurrence risk. The TP63 mutations show rather complete penetrance but highly variable expressivity.^[4]^ Thirdly, In some SHFM cases the conventional karyotyping is sufficient for diagnosis, as it can reveal a large chromosomal aberrations, such as deletions or translocations involving the 7q21-q22 region,^[4,32,33]^ namely SHFM1 locus^[34]^ or deletions of 2q31 (SHFM5).^[34,35]^ Thus, basic GTG banding is still useful and close to molecular testing, as it is a relatively cheap and informative assay in a subset of patients linked to SHFM1 or SHFM5 loci. The important advantage of karyotyping in reference to aCGH is its ability to detect balanced chromosomal rearrangements, for instance translocations, which are not infrequent in SHFM1 locus.

When a clear autosomal recessive inheritance pattern is apparent in non-syndromal SHFM, or when a more common etiology, including an autosomal dominant form associated with germline mosaicism, has been excluded and autosomal recessive inheritance is possible. Then molecular analysis of the WNT10B and DLX5 gene sequencing should be considered. Discovery of SHFM6 locus recently which contains the WNT10B gene,^[29]^ SHFM6-dependent defect is inherited in an autosomal recessive manner and is caused by either homozygous or heterozygous compound of WNT10B mutations. Other autosomal recessive changes originating from SHFM were recently associated with a single family in homozygous DLX5 mutations in the homeodomain of the gene.^[36]^

In such a setting the recurrence risk for the proband's sib is very low, while for the proband's offspring is high and reaches 30% to 50%.^[37]^ Therefore, to ensure healthy reproduction of the next generation, clinical genetic counseling and guidance is of great significance especially for the families of SHFM patients who in need of fertility. First, it helps the affected or at risk individuals to understand the natural genetics of the malformation, and provide them with preventive, supportive measures and clinical management.

Second, it provides a reliable technical guarantee for the actual risk estimation, which allows for a conscious family planning as well as prenatal or pre-implantation diagnosis. For example, for pre-implantation genetic diagnosis of SHFM-affected families with known pathogenic genes, 1 must analyze the cases and draw up genetic testing schemes for couples or other family members. This is followed by preparation of probes for specific pathogenic, genetic genes of patients’ families and establish laboratory genetic diagnosis methods. Then monitor and regulate the ovulation cycle to achieve fertilization in vitro, and continue to culture fertilized eggs into early embryos followed by gaining more embryonic cells through biopsy for genetic diagnosis and testing. Finally, the embryos carrying disease-causing genes are abandoned followed by the unaffected ones that are transferred appropriately and timely. pre-implantation genetic diagnosis is suitable for all SHFM patients or genes carriers who are known to have disease-causing genes. For SHFM patients or carriers with known pathogenic genes, collection and isolation of fetal cells, such as villi, amniotic fluid, and umbilical cord, for genetic diagnosis in the first or second trimester of pregnancy can determine whether the embryo or fetus carries the pathogenic genes, this will help the parents to decide whether to terminate or keep the pregnancy. As significant diagnostic method of reproductive health, prenatal genetic diagnosis is still an important way of preventing and treatment of genetic diseases.

Finally, the exclusion of possible causative alterations, prenatal diagnosis and genetic counseling creates an opportunity for the affected or individual at risk with fertility requirements, that reduces the cost of congenital and developmental abnormalities.

SHFM exists many non-syndromal and syndromal forms which is truly complex from both the clinical and genetically standpoints. Future studies on malformation will improve the ability of Obstetrician and Gynecologist to make better clinical and genetic diagnosis, hence providing a proper prenatal genetic counseling and guidance, then formulate a sound family planning.

## Acknowledgment

The authors would like to thank the patient for allowing this case to be published.

## Author contributions

**Conceptualization:** Shaoyang Lai, Xueqin Zhang.

**Data curation:** Mengzhou He.

**Formal analysis:** Xueqin Zhang.

**Investigation:** Shaoyang Lai, Mengzhou He.

**Methodology:** Mengzhou He.

**Project administration:** Shaoshuai Wang.

**Resources:** Shaoshuai Wang, Ling Feng.

**Software:** Shaoyang Lai, Mengzhou He.

**Supervision:** Ling Feng.

**Validation:** Ling Feng.

**Visualization:** Xueqin Zhang.

**Writing – original draft:** Shaoyang Lai.

**Writing – review and editing:** Shaoshuai Wang.
